# The Mediating Effect of Parenting Stress on the Relationship Between Social Support and Quality of Life in Parents of Children With Autistic Spectrum Disorder: A Meta-Analytic Structural Equation Modeling

**DOI:** 10.3389/fpsyt.2022.713620

**Published:** 2022-02-17

**Authors:** Zhidan Wang, Lin Wang, Siyu Chang, Haijing Wang

**Affiliations:** School of Education Science, Jiangsu Normal University, Xuzhou, China

**Keywords:** autistic spectrum disorder, parenting stress, social support, quality of life, MASEM

## Abstract

**Objective:**

The aim of the present study is to investigate whether parenting stress mediate the relationship between social support and quality of life in parents of children with Autistic Spectrum Disorder. In addition, we examined what other variables *moderate* the relationships in this mediation model.

**Methods:**

Using the two-stage meta-analytic structural equation modeling approach (MASEM), 44 correlation matrices were synthesized from 28 empirical studies (*N* = 13,270) and fitted to the hypothesized mediation model.

**Results:**

There is a significant partial mediation effect of parenting stress on the relationship between social support and quality of life. Subgroup analysis through the first stage analysis suggested that social support measurements, parental role, and child's age moderated the relationship between social support and parenting stress, and that the focus of quality of life moderated the relationship between social support and quality of life. Subgroup analysis through the second stage analysis indicated that parenting stress had a significantly stronger predictive effect on quality of life in Western culture, while the predictive effect of social support on quality of life was significantly stronger in Eastern culture.

**Conclusion:**

Having more social support can reduce parents' stress and then improving their quality of life, which can help them cope more positively and effectively with their autistic children.

## Highlights

- There is a significant partial mediation effect of parenting stress on the relationship between social support and quality of life.
- Social support measurements, parental role, and child's age moderated the relationship between social support and parenting stress.
- Child's age moderated the relationship between social support and parenting stress.
- The focus of quality of life moderated the relationship between social support and quality of life.
- Parenting stress had a significantly stronger predictive effect on quality of life in Western culture, while the predictive effect of social support on quality of life was significantly stronger in Eastern culture.

## Introduction

Autism spectrum disorder (ASD) is a neurological and developmental disorder that starts in childhood and lasts the rest of one's life. Autism is defined by social and linguistic deficits, as well as stereotyped, repetitive behaviors and interests (1). It has an impact on how a person acts and interacts with others, as well as how they communicate and learn. Autism spectrum disorder affects about 1% of the world's population (2). Autism is the most rapidly growing developmental disability. From 2000 (1 in 150) to 2010, the prevalence of autism in children in the United States increased by 119.4 percent (1 in 68) (2). Recently, the prevalence is estimated to be one in every 54 births in the United States (3). According to the first Chinese autistic industry report, published in 2014, the prevalence of autistic children in China is about 1:100, so it is estimated that the total population of autism in China is about 10 million, and the number of autistic children between the ages of 0 and 14 years old is about 2.2 million, growing by 200,000 per year.

Parents of autistic children are vital members of their autistic children's health teams and serve as their autistic children's primary nurses (4). They may face more caregiving challenges than average parents, such as greater treatment expenditures, childcare difficulties, accessing therapeutic facilities that are fairly priced owing to a lack of clinical resources and governmental help (5), and maintaining their socioeconomic level (6). Furthermore, their failure to meet parenting tasks can have an impact on the parents' physiological and cognitive performance, which, when combined with behavioral changes, might hinder them from executing their parental duties effectively (7, 8). Many studies show that parents with ASD children experience greater stress (9, 10) and have a lower quality of life (11). Understanding how parents with ASD children manage with stress and retain a high quality of life is thus an intriguing issue.

### Quality of Life in Families With ASD Children

The WHO defines quality of life (QoL) as ‘the subjective evaluation of one's place in life in the context of one's personal objectives and value systems' (12). Living with an autistic person impacts the entire family, including parents, siblings, and, in certain cases, grandparents, aunts, uncles, and cousins. Meeting the complex demands of a person with an ASD may put families under a lot of emotional, financial, and even physical stress. A consistent finding has been that parents of children diagnosed with ASD are under greater pressure than parents of typical children or of children with other intellectual or developmental disorders (8, 13–17). Researchers have also indicated that the greater the quality of life parents of children with autistic spectrum disorder (ASD) have, the more prepared and supportive they are for dealing with their diagnosed children (18–20). Therefore, it is importance to explore the factors affecting the quality of life of the parents of children with ASD, because improving it can help them cope more effectively and positively with their children.

### The Role of Social Support in Families With ASD Children to Maintain the Quality of Life

Recently, increasing numbers of researchers have identified *social support* as essential for quality of life (20–25). Social support is the help that an individual perceives or obtains from others or from social networks (26, 27). It can be physical or material assistance, or emotional or psychological support (24). According to Cohen and Wills (28) social support theory, social support provides individuals with stable and positive experiences and can affect their physical and mental health. Specifically, social support can effectively regulate an individual's behavior, helping them avoid bad behaviors, form healthy living behaviors, maintain a positive life attitude, and provide predictability, stability, and self-control.

Previous research has suggested that parental stress levels may be influenced by perceptions of social support, particularly for parents of children with autism spectrum disorders (ASD). A lack of social support has been found as the strongest predictor of sadness and anxiety among moms of autistic children (24). Higher levels of social support have been linked to reduced levels of the negative effects of raising an autistic kid, such as psychological discomfort, negative mood, and depressive symptoms (29). Pozo et al. (30) showed that parents of children with ASD who perceived themselves as having more social support also had higher quality of life scores (30). Kuhlthau et al. (31) found that lack of a social support system significantly affects the quality of life of parents of children with ASD. Although many studies have explored the relationship between social support and quality of life in parents of children with ASD, few have further explored whether there are factors that mediate this relationship.

### Parenting Stress With ASD Children

Parental stress is a sort of stress that occurs when a parent's sense of the duties of parenting exceeds his or her resources (32). The emphasis of hassle-focused theories is on the multiple mild to moderate stresses that occur in a regular day or week in the lives of a home with young children or teenagers. Relationship-focused theories emphasize parental stress caused by a variety of factors, including the parent herself or himself, the kid, and the characteristics of their dyadic relationship. Beyond these broad categories, research indicates that parental age, gender, psychopathology symptoms, personality characteristics, and social cognitions (e.g., attitudes, self-concept)—as well as child factors such as serious illness or disability, behavioral and emotional problems or disorders, and typical variations in temperament—all contribute to and are influenced by the level of parenting stress in the caregiver (33).

A large number of researches has been shown that parents of children with autism spectrum disorder (ASD) face significant levels of parental stress, which is connected with bad family outcomes. DesChamps et al. (34) conducted multilevel model analyses to discover that parents of children with ASD concerns had consistently greater levels of parental stress during early child development than parents of children with non-ASD developmental issues or no worries. Some studies have discussed the effects of *parenting stress* on quality of life (20, 23, 35). Hsiao (20) reported parenting stress as a negative predictor of parents' mental quality of life. Dardas and Ahmad (35) found that parenting distress and difficult children—two subscales of parenting stress for fathers of children diagnosed with ASD—are negative predictors of their quality of life.

### The Possible Mediate Role of Parenting Stress Between Social Support and Quality of Life

Because social support has a positive influence on quality of life but parental stress has a negative effect, it's plausible that parenting stress prevents social support from having a positive effect on quality of life. Theoretical research supports the hypothesis that parental stress acts as a moderator in the association between social support and quality of life. For parents with ASD children, a better social support system, one including both informal and formal networks, reduces the negative effects of parenting stress and improves their quality of life (25, 36–39). By contrast, lower levels of social support are likely to cause more parenting stress (10, 24, 40, 41), so parenting stress will further reduce quality of life, stimulating multiple negative emotions in parents and deteriorating spousal and parent-child relationships (8, 42–44).

### Role of Moderator Variables

#### The Role of Parents

Dabrowska and Pisula (14) correctly predicted that the level of stress would be higher in parents of children with autism than in parents of children with Down's syndrome or typically developing children when she administered the questionnaire of resources and stress (QRS) to 162 parents of preschool children with autism. She discovered that moms of children with ASD had greater stress than dads, but she did not detect this problem in either the Down's syndrome or the usually developing group. Falk et al. (10) discovered that mothers of children with ASD, as opposed to fathers, often bear the majority of the additional strain associated with parenting a kid with special treatment requirements and had greater levels of parental stress than fathers. As a result, we anticipate the parent's role to be that of a moderator.

#### The age of the Child

Some studies indicated that mothers of children aged 6–12 years (middle childhood) report considerably greater levels of stress than mothers of children aged 2–5 years (preschool). Other research suggests that stress levels are higher among parents of younger children [e.g., (45–47)], or that stress levels increase with age [e.g., (48, 49)]. Furthermore, a recent research by Peters-Scheffer et al. (50) found no link between children's developmental age, mother stress, and child IQ. Researchers can examine the impact of caring for a kid with ASD over time by collecting different stresses associated with a child's transition across critical developmental phases by assessing the role of child age on parenting stress (46–49). As a result of the inconsistent results reported in prior research, we studied whether the age of the kid might be a moderator.

#### Measurement

We looked to see if the difference between these two measurements had a moderate influence. Some research examined parents' social support using the Multidimensional Scale of Perceived Social Support (MSPSS), a three-factor construct and a 12-item questionnaire: Family, Friends and Significant Other [e.g., (30, 40, 41, 51)]. Other research examined this variable using the Family Support Scale (FSS), a four-factor construct and an 18-item questionnaire: Support for spouses/partners, social organizations, formal kinship, and professional services (39, 52, 53). It is likely that measuring disparities impact social support as well.

#### The Emphasis Is on Quality of Life

Some research concentrated on health-related quality of life (20, 40), while others focused on family quality of life (24, 30, 43). In contrast to quality of life, which is a very wide word, health-related quality of life (HrQoL) refers to those aspects of life that are thought to be influenced by a particular illness (54). Family quality of life (FQoL) is concerned with how individuals perceive their personal quality of life within the family environment, as well as how the family as a whole has opportunities to pursue its significant potential and achieve its objectives in the community and society of which it is a part (55). As a result, we investigated whether the researchers' emphasis on various areas of life quality would have a mild influence.

#### Cultural Context

According to research, there are consistent cultural differences in how individuals regard themselves and their relationships, which may have an impact on whether or not they use social support to cope with stress. Westerners see people as autonomous and apart from others, but Asians see people as intrinsically related to others (56–58). Because Asians place a premium on connection with their social group, this distinction may lead to the idea that coping through social support is more widespread among them. Taylor et al. (59), on the other hand, found that Asian and Asian American students reported using social support much less for coping with stressful situations than European American students, a pattern that was especially apparent for Asian nationals and first-generation students. Furthermore, a subsequent research found that relational considerations (e.g., a desire to maintain group unity and anxiety that revealing issues might result in unfavorable appraisal by others) entirely moderated the relationship between culture and non-use of social assistance for stress management. Because of the contradictory results reported in prior studies, we studied whether cultural background might act as a moderator.

### The Present Study

As previous empirical studies based on multifactor analysis and linear regress analysis has not drawn reliable conclusions, it is necessary to use meta-analytic structural equation modeling (MASEM), a new approach that can integrate multiple findings using different analysis methods to explore further. MASEM allows not only for testing of the theoretical framework, but for reducing or even eliminating measurement and sampling errors from a single research finding, thus improving the external validity (60). The main goal of this study was to investigate the relationships among social support, parenting stress, and quality of life. Specifically, we hypothesized that *parenting stress* is likely to be a mediator between *social support* and *quality of life*. It should be noted that this study is among the ***first*** to explore the relationships among social support, parenting stress, and quality of life of parents of children with ASD using the MASEM approach. The secondary goal of this study was to examine whether other variables *moderate* the relationships in this mediation model. This is an essential issue to investigate since increasing the quality of life for parents who have children with ASD is critical to reducing stress and sustaining mental health.

## Methods

### Ethical Statement

This study did not involve human participants, thus, the informed consent was not needed. The study was reviewed and approved by the ethnical committee of [Blinded] University.

### Search Procedures

**Figure 1:**
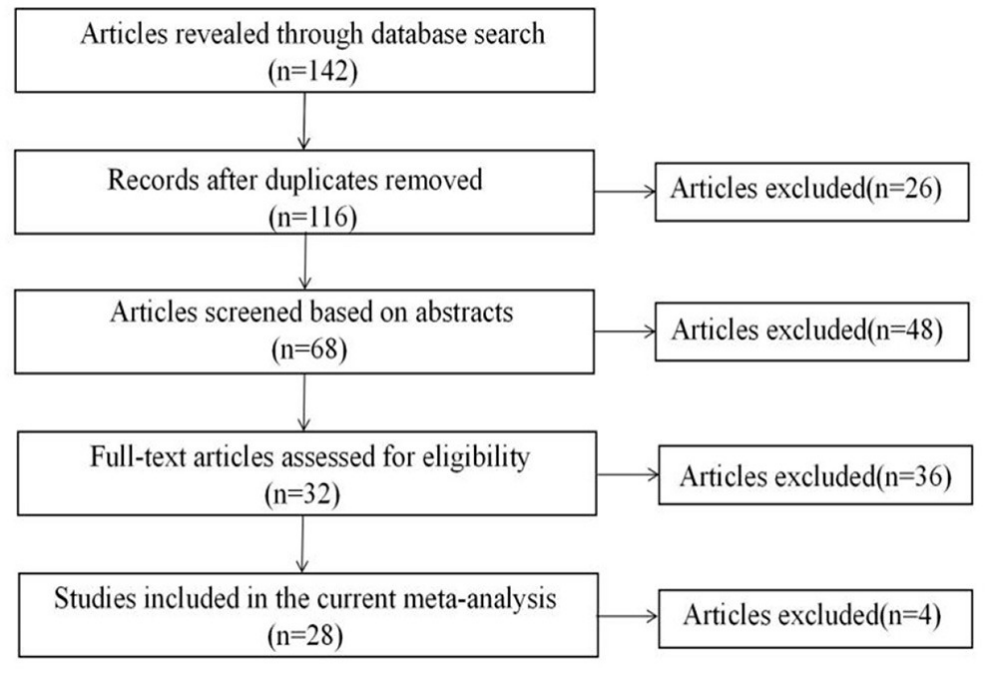
Flowchart of the search and screening process.

In order to provide a representative literature base, a systematic search process that is consistent with the recommendations of the previous researchers and has been applied by previous meta-analyses [e.g., (61, 62)] was used. An overview of search and screening procedures is presented in Figure 1. First, computerized searches was conducted in the English databases Google Scholars, Emerald, Science Direct, Wiley, Springer Link, Taylor and Francis, SAGE Journals, PubMed, Scopus, Web of science, EBSCOhost, ERIC and Chinese databases China National Knowledge Infrastructure (CNKI) from January 1, 2010 to December 31, 2021. Search strategies included keywords pertaining to ASD (including “autis^*^, “Asperger,” and “pervasive developmental disorder”) [e.g., (61)]. These terms were combined with terms “parenting stress,” “social support,” and “quality of life.” Second, manual searches in the most important academic journals about autism spectrum disorder (ASD) (e.g., *Journal of autism and developmental disorders, Journal of Developmental and Physical Disabilities*) as well as in conference proceedings were conducted. Third, the references of identified articles and major reviews on related topics were examined to search for missed studies. Additionally, working papers and unpublished dissertations were searched to avoid potential publication bias. The initial search yielded 142 records.

### Inclusion Criteria

Studies were eligible for the meta-analysis database on the basis of several criteria. First, only empirical studies that simultaneously measured any two variables among parenting stress, social support, and quality of life were included. Second, studies had to report at least one Pearsons's correlation coefficient (*r*) between the dimensions, or a total score of one variable and the dimensions or total score of another variable. Third, studies needed to focus on the parents of children between 0 and 8 years old with ASD. Fourth, only studies which used the same concept or definition of the variables mentioned above were included to ensure meaningful comparability. Fifth, the studies' sample sizes had to be clear. Sixth, for those studies that reported duplicate data, the most comprehensive one was selected.

### Exclusion Criteria

Samples in which the participants were not the parents of children with ASD and for using qualitative data only were excluded. Studies were excluded if the parents of the sample had children who were at-risk for ASD without a formal diagnosis or lived away from the family home.

### Study Selection and Data Extraction

Initially, studies were reviewed for inclusion by WL and CS. They were agreed on the majority of the studies (89.2%). The disagreements (3 studies) were discussed and resolved by CS and WH. Then, data were extracted by WL and CS. Discrepancies in data extraction were resolved by consensus (CS and WH).

Based on the above criteria, 26 studies were excluded via reading the titles, because they were duplicates. Forty eight studies were excluded because they were not empirical, as determined by reading the abstracts. Thirty six studies were excluded because Pearsons's correlations of interest were not reported, as determined by reading the full text. Four studies were excluded because the children were over 18 years old. As a result, 28 studies involving 31 independent samples and 44 correlations were identified for the final meta-analysis (Figure 1).

### Coding

The studies were coded in three steps [e.g., (63, 64)]. First, each study was coded for the following information: study name, sample size, available effect size, parents' role, measurements of social support, and focus on quality of life and sociocultural background. The parents' role from original samples was coded as “mother” or “father.” The measurements of social support were coded as measured by (FSS) or by the Multidimensional Scale of Perceived Social Support (MSPSS). The sociocultural background was coded as “east” or “west.” The focus on quality of life was coded as “family quality of life” (FQoL) and “health-related quality of life” (HQoL). Table 1 presents the definitions and their representative operationalizations of the key variables included in this study (Table 1).

**Table 1:** Definition and examples of key variables.

**Variable**	**Definition**	**Operationalization/example**
Social support	Social support refers to the help that an individual perceives or actually obtains from others or social network.	Perceived social support (e.g., (23)); Social support rating scale (SSRS) from Xiao (65); Family support [e.g., (66)];
Parenting stress	Parenting stress refers to the negative psychological reaction to the various demands of being a parent.	Parental stress (e.g., (43) and (67)); Parenting Stress Index-Short Form (PSI-SF) from Abidin (68); Caregiver Burden Inventory from Chou et al. (69)
Quality of life	Quality of life refers to one' s perception of their own life status under a specific culture and value system they live.	Health-related quality of life [e.g., (40)]; Mental health-related quality of life [e.g., (20)]; Family quality of life [e.g., (30)]; World Health Organization Quality of Life Brief Scale (WHOQOL-BREF) from WHOQOL Group (70)

Second, the following principles were abided by when coding: (i) the extraction of effect size was based on independent samples; (ii) for the cases in which an independent sample was reported with one correlation coefficient among social support, parenting stress, and quality of life, the coding was conducted once; (iii) for the cases in which an independent sample provided more than one correlation coefficient (such as a correlation coefficient not only between social support and parenting stress, but also between social support and quality of life) the coding was conducted individually for each correlation relationship; (iv) for the cases in which an independent sample only reported a Pearson's correlation matrix between the subdimensions of two variables, the formula:


$$\begin{array}{l} {\text{r}}_{\text{xy}}=\frac{\sum r_{xi}r_{yi}}{\sqrt{n+n\left(n-1\right){\bar{r}}_{xixj}}\sqrt{m+m\left(m-1\right){\bar{r}}_{yiyj}}} \end{array}$$

was used to synthesize the overall correlation coefficient for coding; and (v) for those studies that were longitudinally designed, the first outcome was chosen for coding.

Finally, one researcher coded the variables and information as above and another researcher independently coded a randomly selected sample of two thirds of the studies. Inter-rater agreement calculated as [agreements (agreements + disagreements) ×100] reached a high level for all codings (above 90% throughout) (71). Discrepancies between two coders were solved through discussions regarding the original studies. This yielded a final database of 44 effect sizes reported in 28 studies, with a total sample size of 13,270 (see Table 2 for basic information about the 44 correlations).

**Table 2:** Basic information from the original studies for the meta-analysis research.

**References**	**Publication status**	**Sociocultural background**	**Child age**	**N**	**Role of parents**	**Relationship**	***r***	**SS** **measurement**	**QoL focus**
Akram et al. (72)	Published	East	NR	339	Mother	SS-PS	−0.53	MSPSS	NI
Ban and Sun (73)	Published	East	NR	245	Both	SS-PS	−0.238	Other	NI
Bohadana et al. (74)	Published	West	8.90	139	Both	SS-PS	−0.53	MSPSS	NI
						SS-QoL	0.62	MSPSS	OQoL
Chu et al. (21)	Published	East	NR	110	Both	PS-QoL	−0.57	NI	OQoL
Drogomyretska et al. (75)	Published	West	8.17	454	Nr	SS-PS	−0.133	MSPSS	NI
Falk et al. (10)	Published	West	8.38	229	Father	SS-PS	−0.26	Other	NI
				250	Mother	SS-PS	−0.23	Other	NI
Hall and Graff (52)	Published	West	8.00	73	Both	SS-PS	−0.252	FSS	NI
Henry (22)	Unpublished	West	NR	115	Mother	PS-QoL	−0.743	NI	OQoL
Gu (76)	Unpublished	East	NR	115	Both	SS-PS	−0.512	Other	NI
Hsiao (20)	Published	West	NR	429	Nr	SS-PS	−0.155	Other	NI
						SS-QoL	0.374	Other	HQoL
						PS-QoL	−0.33	NI	HQoL
Hsiao (43)	Published	West	NR	236	Both	PS-QoL	−0.494	NI	FQoL
Khanna (40)	Unpublished	West	NR	304	Both	SS-PS	−0.38	MSPPS	NI
						SS-QoL	0.391	MSPPS	HQoL
						PS-QoL	−0.472	NI	HQoL
Kuru and Piyal (23)	Published	West	NR	90	Both	SS-QoL	0.524	MSPPS	OQoL
Lei and Kantor (24)	Published	East	NR	163	Both	SS-QoL	0.424	Other	FQoL
Li (77)	Unpublished	East	NR	211	Nr	SS-QoL	0.407	Other	FQoL
Liu et al. (78)	Published	East	4.75	95	Mother	SS-PS	−0.561	Other	NI
Liu (79)	Unpublished	East	5.74	1,384	Both	SS-PS	−0.26	FSS	NI
						SS-QoL	0.44	FSS	FQoL
						PS-QoL	−0.49	NI	FQoL
Lu et al. (27)	Published	East	6.68	479	Both	SS-PS	−0.322	MSPPS	NI
Pozo et al. (30)	Published	West	12.40	118	Mother	SS-QoL	0.296	Other	FQoL
Rutstein (53)	Unpublished	West	4.30	25	Mother	SS-PS	−0.61	FSS	NI
Singh et al. (51)	Published	East	8.01	70	Mother	SS-PS	−0.481	MSPPS	NI
Tomeny (67)	Unpublished	West	12.03	115	Both	SS-PS	−0.15	Other	NI
Wang et al. (44)	Published	East	5.15	150	Mother	SS-PS	−0.16	Other	NI
Wang (80)	Unpublished	East	4.66	625	Both	SS-QoL	0.52	Other	FQoL
Weinberg et al. (81)	Published	East	9.36	105	Both	SS-PS	−0.48	MSPPS	NI
			6.80	104	Both	SS-PS	−0.35	MSPPS	NI
Zaidman et al. (38)	Published	West	3.24	283	Mother	SS-PS	−0.57	Other	NI
Zeng et al. (25)	Published	East	10.30	219	Mother	SS-PS	−0.237	FSS	NI
						SS-QoL	0.533	FSS	FQoL
						PS-QoL	−0.381	NI	FQoL
				219	Father	SS-PS	−0.239	FSS	NI
						SS-QoL	0.594	FSS	FQoL
						PS-QoL	−0.360	NI	FQoL
Zeng et al. (39)	Published	East	10.30	226	NR	SS-PS	−0.238	FSS	NI
						SS-QoL	0.531	FSS	FQoL
						PS-QoL	−0.384	NI	FQoL

*The same study contains two independent samples by adding 01 and 02 after the age to distinguish. r, correlation coefficient; SS, social support; PS, parenting pressure; QoL, quality of life; FQoL, family quality of life; HQoL, health-related quality of life; OQoL, overall quality of life; NI, not involved; NR, not reported; MSPPS, Multidimensional Scale of Perceived Social Support; FSS, Family Support Scale*.

### Methods of Analysis

The present study adopted the two-stage meta-analytic structural equation modeling (MASEM) approach to incorporating meta-analytic techniques, under the general framework of structural equation modeling (SEM) (82). Stage one pooled correlation matrices together from primary studies using Comprehensive Meta-Analysis Version 3.3 (CMA 3.3). Stage two fitted the pooled correlation matrix to estimate a hypothesized structural equation model using IBM SPSS Amos 24.0 .

#### Stage One Analysis

Stage One analysis included creating a correlation matrix and evaluating moderation effects. First, the meta-analytic techniques most commonly applied in current ASD research [e.g., (61, 83)] were used to create a correlation matrix. Prior to analysis, measurement error was corrected through dividing the correlation coefficient by the product of the square root of the reliabilities of the two constructs. To account for varying sample sizes in the studies, the effect size of each study was weighted using the inverse variance weight and then reconverted into correlation coefficients (84). Specifically, the effect sizes (R) were transformed into Fisher's *Z*-coefficients using sample size as weighted value, *Z* $=\;0.5\times \ln\left(\frac{1+R}{1-R}\right)$ (84). Then, the average Z after conversion was calculated and finally reconverted to the R value, *R* = $\frac{e^{2Z}-1}{\;e^{2Z}+1}$. The heterogeneity test involving checking the Q and *I*^2^values was used to determine the final effect sizes in the correlation matrix. *I*^2^values of ~25, ~50, and ~75% were interpreted as low, moderate, and high, respectively (85). When the Q test was significant or the *I*^2^values were higher than 75%, the random effects model was more appropriate and variability was attributed from both within-study and between-study variance. Otherwise, the fixed effects model was chosen and variability was only stemmed from within-study variance (86). Then, classic Fail-safe N was used to check for publication bias. When the Fail-safe number was >100, there was sufficient evidence to prove that there was no publication bias in our meta-analysis (87). The main relationships in the correlation matrix were built on a minimum of at least seven samples, which is in line with other MASEM studies (88, 89).

After the correlation matrix was finished, subgroup analysis was used to evaluate moderation effects when heterogeneity was significant (90). A meta-variance-analysis step was conducted to determine whether there was a significant difference between each subgroup's average effect size (91). As discussed in the literature section, the parents' roles, measurements of social support, focus on quality of life, and sociocultural backgrounds were used as potential moderators in order to assess their abilities to explain between-study variance.

#### Stage Two Analysis

Stage Two analysis included examining the partial mediation model. Prior to analysis, reliability for a single variable was set to the average value of the reliability of all scales assessed for that variable from original studies, and for the variables not reported with its scales' reliability. In one original study, 0.8 was set as a conservative estimate for its reliability (92). The measurement error of the observation model corresponding to each variable is 1 minus the average reliability value of that variable. As the relationships were based on different sample sizes, the recommendation to use the harmonic mean of the sample sizes across all cells for path analysis was followed (93, 94). In analysis, the correlation matrix was analyzed path-analytically using maximum likelihood estimation procedures. The Monte Carlo (parametric bootstrap) method was used to test the mediation effect. This method does not require original data, so it can be applied to analyze mediation effect by using only a correlation matrix or a covariance matrix (95). Then, the mediation effect hypothesis was further examined by comparison of the saturated partial-mediation model and the full-mediation model. The former includes direct effect of social support on quality of life while the latter omits it. In a saturated partial-mediation model, the number of observed variables is equal to the number of parameters, so that the saturated model has zero degrees of freedom. Therefore, although the model can reproduce the observed correlation/variance-covariance matrix perfectly, measures of model fit cannot be applied.

## Results

### Meta-Analysis

Table 3 provides the weighted correlations between (1) parenting stress and social support, (2) parenting stress and quality of life, (3) social support and quality of life. As previously described, the heterogeneity of three weighted average correlations were assessed using Q statistics. Q statistics showed most analyses had significant between-study heterogeneity (*p* <0.001) (see Table 3), so the random effects model was chosen to estimate the three weighted correlations. Specifically, the weighted average correlation between parenting stress and social support was −0.339 (k = 23; 95% CI [0.398, −0.277]), indicating a moderately strong negative relationship; The weighted average correlation between parenting stress and quality of life was −0.470 (k = 9; 95% CI [−0.537, −0.396]), indicating a moderately strong negative relationship; The weighted average correlation between social support and quality of life was 0.474 (k = 12; 95% CI [0.425, 0.520]), indicating a moderately strong positive relationship. In addition, classic fail-safe N was checked to estimate whether the unpublished and unacquired studies would has an impact on our meta-analysis outcomes. Findings showed that the fail-safe number of each effect size is greater than 100 (see Table 3), indicating that there is no publication bias in our meta-analysis (87).

**Table 3:** Analysis of correlation.

**Relationship**	**K**	**N**	***R***	**95% interval**		***I* ^2^**	**Fail-safe N**
				**Lower limit**	**Upper limit**		
SS-PS	23	6,051	−0.339***	−0.398	−0.277	84.156***	3,437
PS-QoL	9	3,242	−0.470***	−0.537	−0.396	82.042***	1,588
SS-QoL	12	4,127	0.474***	0.425	0.520	69.490***	2,800

SS, social support; PS, parenting stress; QoL, quality of life; K, independent samples; N, sample size; R, weighted average correlation; ^***^p <0.001.

### Subgroup Analysis

Table 4 provides subsequent moderator analyses. (1) Focus of quality of life was identified as a significant moderator (Q = 5.558, *p* < 0.005). The correlation between social support and quality of life tended to be stronger when the focus of quality of life was family (FQoL) (*R* = 0.479, k = 8) compared to that was health (HQoL) (*R* = 0.381, k = 2). (2) A significant moderating effect of social support measure was found (Q = 5.482, *p* < 0.05). The correlation between social support and parenting stress tended to be stronger when the MSPSS (*R* = −0.401, k = 8) was used to measure social support compared with the FSS (*R* = −0.257, k = 6). (3) Role of parents was identified as a significant moderator (Q = 4.438, *p* < 0.05). The relationship between social support and parenting stress tended to be stronger among mothers (*R* = −0.423, k = 8) than among fathers (*R* = −0.250, k = 2). (4) Child's age was identified as a significant moderator between social support and parenting stress (Z = 2.31, *p* = 0.021). As children grow older, the correlation between social support and parenting stress becomes stronger (b = 0.037, k = 18). (5) Sociocultural background was not found to be a moderating factor among three relationships.

**Table 4:** Analysis of moderation.

**Relationship**	**Categorical Moderator**	**Level**	**K**	***R***	**95% interval**		**Q-value**
					**Lower limit**	**Upper limit**	
PS-QoL	Sociocultural background	East	5	−0.438	−0.507	−0.363	0.815
		West	4	−0.518	−0.652	−0.351	
SS-QoL	Focus of QoL	FQoL	8	0.479	0.424	0.530	5.558*
		HQoL	2	0.381	0.317	0.441	
	Sociocultural background	East	8	0.487	0.440	0.532	0.621
		West	5	0.442	0.333	0.540	
SS-PS	Measurement	FSS	6	−0.257	−0.296	−0.217	5.482*
		MSPSS	8	−0.401	−0.503	−0.228	
	Role	Father	2	−0.250	−0.335	−0.351	4.438*
		Mother	8	−0.423	−0.542	−0.286	
**Relationship**	**Continuous Moderator**	**P-value**	**K**	***b***	**95% interval**		**Z-value**
					**Lower limit**	**Upper limit**	
SS-PS	Child age	0.021	18	0.037	0.006	0.068	2.31*

K, independent samples; R, weighted average correlation; FSS, Family Support Scale; MSPSS, Multidimensional Scale of Perceived Social Support; FQoL, family quality of life; HQoL, health-related quality of life; ^*^p < 0.05.

### Mediation Effect Analysis

As mentioned, the meta-analytic pooled correlation matrix was next used as input for the structural equation model. The correlation matrix showed that the average reliability of parenting stress, social support and quality of life was respectively 0.860, 0.837 and 0.874 (see Table 5).

**Table 5:** Pooled correlation matrix across studies.

**Variable**	**Parenting stress**	**Social support**	**Quality of life**
Parenting stress	0.860	23	9
Social support	−0.346	0.837	12
Quality of life	−0.470	0.474	0.874

*The diagonal is the reliability of each variable*.

**Figure 2:**
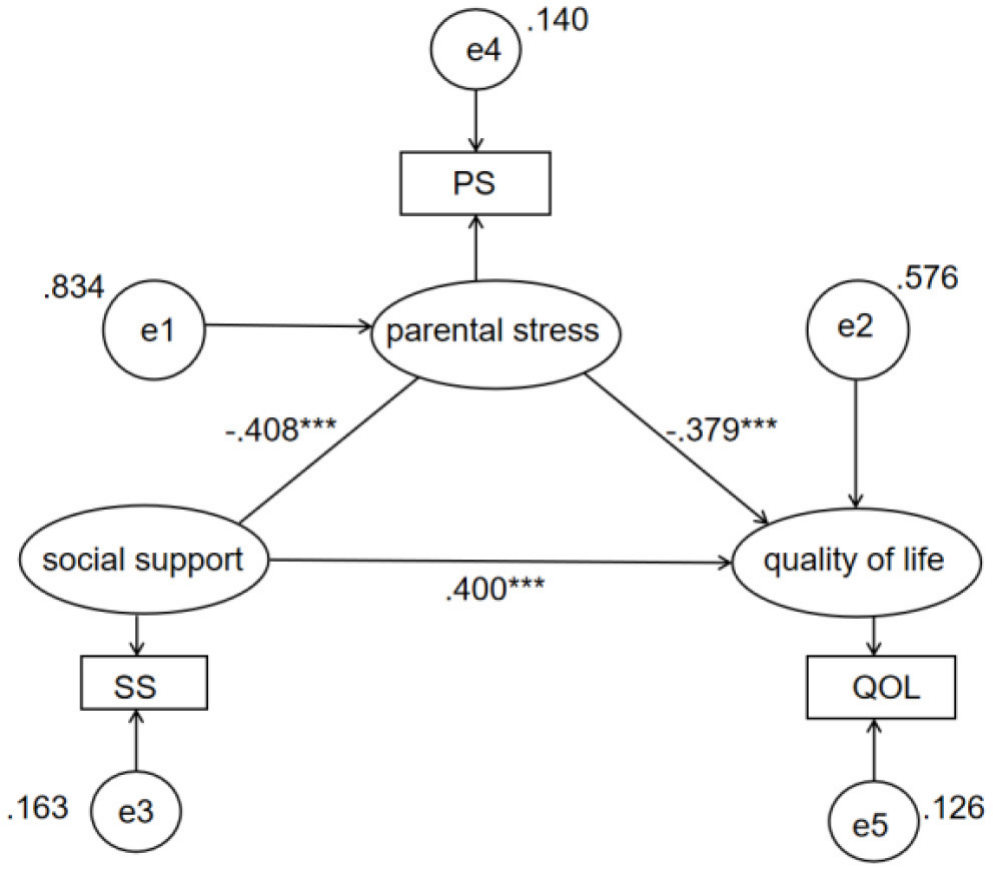
Path analysis of the mediation model.

Then, the measurement error of each observation model corresponding to the three variables was 0.140, 0.163 and 0.126 (see Figure 2). Path analysis findings suggested that the latent factors had relatively good loading coefficients (see Figure 2). Social support significantly predicted parenting stress, β = −0.408, *p* < 0.001 and significantly predicted quality of life, β = 0.400, *p* < 0.001, parenting stress made significant contributions to quality of life, β = −0.379, *p* < 0.001.

In addition, the comparison of different mediation models indicated that the full mediation model (χ^2^ = 530.225, *df* = 1) and the saturated partial mediation model (χ^2^ = 0, *df* = 0) were significantly different, Δχ^2^ = 530.225, *p* < 0.001, and the former exhibited significantly poor fit (RMSEA = 0.357), thus supporting the partial mediation model, in which both the direct and the indirect effect of social support on quality of life were significant (direct effect = 0.400, *p* < 0.001; indirect effect = 0.155, total effect = 0.554; *p* < 0.001) (see Table 6).

**Table 6:** Mediation effect analysis.

				**Monte Carlo**	
		**Product of coefficient**		**Percentile 95% CI**	
**Effect**	**Estimates**	**SE**	**Z**	**Lower**	**Upper**
Indirect effect	0.155**	0.009	17.333	0.138	0.174
Direct effect	0.400**	0.017	23.176	0.358	0.425
Total effect	0.554**	0.016	34.375	0.517	0.578

*p < 0.05, ^**^p < 0.01, ^***^p < 0.001.

### Group Comparison

Finally, sociocultural background was chosen to further explain between-study heterogeneity by structural equation modeling (see Table 7).

**Table 7:** Pooled correlation matrix under different sociocultural background.

**Variable**	**Parenting stress**	**Social support**	**Quality of life**
Parenting stress	-	−0.324	−0.518
Social support	−0.351	-	0.442
Quality of life	−0.438	0.493	-

*The eastern group data is at the bottom left of the correlation matrix (15 studies, 8,955 participants, harmonized N = 2,834); The western group data is at the top right of the correlation matrix (13 studies, 4,465 participants, harmonized N = 1,314)*.

**Figure 3:**
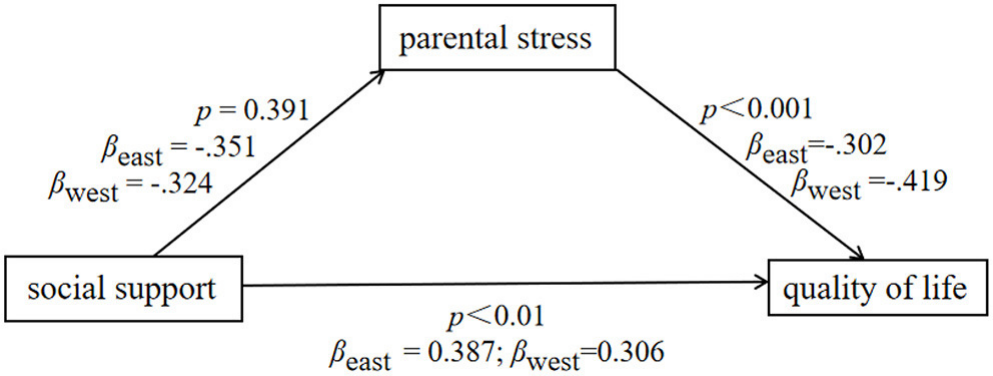
Path comparison.

The group comparison findings suggested that the predictive effect from social support to parenting stress in the eastern background (β = −0.351) was not significantly different from that in the western background (β = −0.324), CMIN = 0.736, *p* = 0.391; the predictive effect from parenting stress to quality of life in the western background (β = −0.419) was significantly stronger than that in the eastern background (β = −0.302), CMIN = 16.447, *p*< *0.0*01; the predictive effect from social support to quality of life in the eastern background (β = 0.387) was significantly stronger than that in the western background (β = 0.306), CMIN = 7.878, *p*< *0.0*1 (see Figure 3). Overall, it is suggested that sociocultural background significantly explained the between-study heterogeneity of the latter two paths.

## Discussion

The main goal of this study was to explore the relationships among social support, parenting stress, and quality of life of parents of children with ASD. The first hypothesis, that parenting stress was a mediator between social support and quality of life, was supported. It was found through meta-analysis that social support and quality of life had a moderately significant positive correlation (*r* = 0.474), and the conclusion that social support can directly predict quality of life among parents of children with ASD was consistent with the findings of Bohadana et al. (74), Hsiao (20), Khanna (40), and Pozo et al.'s (30) studies. In addition, on the basis of a large number of independent research findings from multiple contexts, it was found through mediating effect analysis that parenting stress played a partially mediating role between social support and quality of life.

The secondary goal of this study was to examine whether other variables *moderate* the relationships in this mediation model. First, parents' role is a moderator that affects parenting stress and social support among the parents of children with ASD. When the role is mother, the negative correlation coefficient between social support and parenting stress is higher than when the role is father. Mothers take on more tasks of caring for children with ASD and deal more with the children's emotional and behavioral problems in daily life (43). In addition, mothers are more inclined to attribute their children's problems to themselves when their children are affected by illness or disability (10). Therefore, the mothers of children with ASD are likely to show more parenting stress, which further strengthens the relationship between their perceived social support and their parenting stress.

Second, the measurements of social support had a significant moderating effect on social support and parenting stress. The correlation between social support and parenting stress measured by MSPSS was stronger than that measured by FSS. The MSPSS measures the three types of help available to parents: family, friends, and significant others (96); the FSS measures the helpfulness of four types of social support: from informal kinship, spouses or partners, social organizations, formal kinship, and professional services (97). Previous studies have reported that MSPSS had a higher reliability score and was more commonly used than FSS among parents of children with disabilities (23, 51–53, 81, 98). In line with these findings, we showed that the correlation between social support and parenting stress measured by MSPSS was stronger than that measured by FSS.

Third, the focus of quality of life moderated the relationship between social support and quality of life among parents of children with ASD. The correlation between *family quality of life* and social support was greater than that between *health-related quality of life* and social support. The health-related quality of life consists mainly of an individual's physical and mental health status, whereas family quality of life concerns not only the dynamic sense of physical and emotional well-being of the family, but also focuses more on family interaction, parenting, and disability-related support (40, 99). Previous studies have indicated that the highest-satisfaction aspect of quality of life among parents of children with ASD was the family interaction domain, which belongs to the *family quality of life domain* (25, 100, 101), and the lowest was emotional health, which belongs to the health-related quality of life domain (39, 43). Therefore, this moderating effect may be due to the parents' different levels of satisfaction with different aspects of quality of life.

Fourth, we hypothesized that the age of the kid was a significant modulator of social support and parenting stress. The result showed that the link between social support and parenting stress increases stronger as children become older. Consistent with prior findings (45), age and the degree of autism are associated; that is, as age grows, so does the severity of autistic features in social interactions, communication, and flexible thinking. It is probable that the long-term investment of time, energy, and money causes parents with autism to feel more weary and worried than before, and in this instance, the function of social support in reducing parental stress is especially crucial. As a result, the relationship between social support and parental stress has grown stronger as children with ASD age older.

Finally, in the second stage meta-analytic structural equation modeling study, it was established that the mediation effect under eastern sociocultural context differed from that under western sociocultural background. Parenting stress had a significantly stronger predictive effect on quality of life in the western sociocultural background than in the eastern sociocultural background, indicating that parenting stress is a more important internal factor for parents of children with ASD in western sociocultural countries. Meanwhile, the predictive effect of social support on quality of life was significantly stronger in the eastern sociocultural background than in the western sociocultural background, indicating that social support, an important external factor, is more important for parents of children with ASD in eastern countries. These findings might be attributed to cultural differences between the East and the West. Individualism is the norm in the West, and people's relationships are generally autonomous (102), but collectivism is the norm in the East, and people's relationships are more interconnected and interdependent (103). As a result, compared to external factors, internal factors such as parental stress are more directly associated to the quality of life of parents of children with ASD in the West. On the contrary, lack of external social ties, such as social support, is more likely to influence the quality of life of parents of children with ASD in the East. It is suggested that cultural differences should be considered to improve the quality of life among parents of children with ASD.

### Limitations and Future Directions

This study suffers from various limitations that might present numerous chances for future research by taking a first step toward assessing the partial mediation model among social support, parenting stress, and quality of life among parents of children with ASD using MASEM. First, the present study failed to estimate the joint correlation matrix among the sub-dimensions of social support, parenting stress, and family life quality because the measurements used in each original study were different, and most of these studies focused on the links among variables in general rather than the sub-dimensions. Future study on this issue should broaden the nature and extent of existing research, allowing for more in-depth analysis of these intriguing constructs as well as testing the structural validity of each notion. Also, additional qualities such as parental self-efficacy, resilience, and coping mechanisms, according to the buffering hypothesis, may function as mediators to reduce the impact of stressful events on parents of disabled children's quality of life (104–107). However, due to a lack of correlation studies between social support, quality of life, and these factors, it is not viable to investigate further mediation channels using MASEM. As a result, more study is needed in the future to offer a full picture of possibly additional mediating mechanisms, enhancing the buffering effect hypothesis in the field of health issues among parents of children with ASD. Furthermore, the present MASEM study depends on data from previous investigations, which often utilized cross-sectional designs, making causal inferences impossible. As a result, longitudinal designs are urged in future research to better understand the links between social support, parental stress, and quality of life among parents of children with ASD.

### Conclusion

In conclusion, this study used a two-stage MASEM approach to investigate the relationships among social support, parenting stress, and quality of life in parents of children with ASD. On the basis of 28 original studies involving 44 correlations, we found a significant partial mediation effect of parenting stress on social support and quality of life. Subgroup analysis through the first stage analysis suggested that social support measurements, parental role, and child's age moderated the relationship between social support and parenting stress, and that the focus of quality of life moderated the relationship between social support and quality of life. Subgroup analysis through the second stage analysis indicated that the mediation effect under eastern sociocultural background was quite different from that under western sociocultural background.

## Data Availability Statement

The original contributions presented in the study are included in the article/supplementary material, further inquiries can be directed to the corresponding author/s.

## Author Contributions

ZW and LW conceptualized the research. LW, SC, and HW reviewed and coded the data. LW extracted the data. HW and SC solved the discrepancies of the data. LW analyzed the data. ZW, LW, SC, and HW wrote the manuscript.

## Funding

This research was supported by Jiangsu Social Science Fund (18JYC003) to ZW.

## Conflict of Interest

The authors declare that the research was conducted in the absence of any commercial or financial relationships that could be construed as a potential conflict of interest.

## Publisher's Note

All claims expressed in this article are solely those of the authors and do not necessarily represent those of their affiliated organizations, or those of the publisher, the editors and the reviewers. Any product that may be evaluated in this article, or claim that may be made by its manufacturer, is not guaranteed or endorsed by the publisher.
